# Microbial Biodegradation of Chlorothalonil Residual Pollutants in Soil and Tomato Plants by Microencapsulated *Proteus terrae* ZQ02

**DOI:** 10.3390/toxics14050352

**Published:** 2026-04-22

**Authors:** Sajjad Ahmad, Jie Liu, Murugesan Chandrasekaran

**Affiliations:** 1 College of Plant Protection, South China Agriculture University, Guangzhou 510642, China; iamdrsajjad@gmail.com; 2 Department of Food Science and Biotechnology, Sejong University, 209 Neundong-ro, Gwangjin-gu, Seoul 05006, Republic of Korea

**Keywords:** chlorothalonil, residual pollution, microencapsulation, biodegradation, soil

## Abstract

Chlorothalonil is a widely used fungicide in agriculture, but its excessive application can lead to environmental contamination. This study investigated the biodegradation potential of *Proteus terrae* ZQ02 in free and immobilized forms. Under optimal conditions (37 °C, pH 7), free cells degraded 97.2–98.7% of chlorothalonil (50 mg/L) within seven days. Bacterial microcapsules were prepared using 3% sodium alginate, 2% calcium chloride, and 60 g/L wet biomass, with encapsulation times ranging from 6 to 12 h. The microcapsules displayed uniform size, high mechanical strength, porous structure, and excellent mass transfer, ensuring stable degradation activity. Encapsulated cells demonstrate enhanced tolerance to variations in pH, temperature, and salinity compared to free cells. In soil, microcapsules reduced chlorothalonil half-lives to 1.33–5.45 days for concentrations of 10–30 mg/L, achieving 92–96% degradation over 14–35 days. In tomato-planted soils, encapsulated and free cells degraded 96.3% and 81.6% of residues, respectively, after 28 days, significantly exceeding the control. These findings highlight that immobilization improves the stability, reusability, and efficiency of *P. terrae* ZQ02, making it a promising strategy for sustainable chlorothalonil biodegradation. The study demonstrates the potential of combining microbial strains with carrier materials for effective pesticide remediation and environmental protection, providing a foundation for large-scale applications in contaminated agroecosystems.

## 1. Introduction

Since 1950, pesticide development has accelerated significantly and has become a key driver of increased agricultural production. Pesticides are widely used by farmers to control weeds, insect pests, and microbial diseases, thereby enhancing crop yield [1,2]. The continuous growth of the global population would not have been possible without a corresponding increase in food production. It is estimated that pesticides contribute to the production of approximately one-third of agricultural outputs. Without their use, the production of fruits, vegetables, and cereals would decline by 78%, 54%, and 32%, respectively [3]. Therefore, pesticides play a fundamental role in modern crop production.

Chlorothalonil (2,4,5,6-tetrachloro-1,3-benzenedicarbonitrile, CAS 1897-45-6) is a broad-spectrum, non-systemic chlorinated benzonitrile fungicide that was first registered in the USA in 1966. It is extensively used in modern agriculture worldwide to protect more than 50 types of vegetables, fruits, ornamental plants, and turf grasses from fungal diseases, ultimately contributing to increased crop yields for the growing global population [4,5,6]. Additionally, chlorothalonil is extensively used in antifouling paints to prevent marine biofouling on ship surfaces. However, due to its indiscriminate application, chlorothalonil residues are commonly found in both aquatic and terrestrial environments, posing a significant threat to various non-target organisms [7]. Its persistence in the environment is exacerbated by its long half-life in soil and sediments (60–100 days) and low water solubility (<10 mg/L), which allow residues to accumulate for over a year following repeated applications [8]. Studies have shown that chlorothalonil residues are highly toxic to aquatic life, humans, mammals, birds, beneficial soil arthropods, and enzymatic activity in the soil ecosystem [9,10,11,12]. In humans, exposure to chlorothalonil has been linked to severe health issues, including eye and skin irritation, gastrointestinal distress, dermatitis, and even cancer [5,13,14]. Soil microbial ecology was assessed using soil properties and DGGE (Denaturing Gradient Gel Electrophoresis), a technique that evaluates the diversity of microbial communities. The findings demonstrated that residual chlorothalonil significantly affected microbial activity, boosting soil respiration and phosphatase activity while lowering saccharase activity, microbial biomass, and diversity. This knowledge was crucial for understanding microbiological toxicity [15]. Another study has shown that relevant concentrations of chlorothalonil can cause DNA damage, abnormal development, and increased mortality in various marine species [16]. Embryonic development in numerous species and other developmental processes are adversely affected by chlorothalonil. Additionally, it reduces organism survival and has genotoxic effects, including chromosomal abnormalities and altered gene expression [17].

To evaluate the environmental fate of chemicals under control conditions, standardized biodegradation tests developed by organizations such as the International Organization for Standardization (ISO) and the Organization for Economic Co-operation and Development (OECD) are widely employed [18,19]. The OECD framework comprises a three-tiered system of ready, inherent, and simulation tests, which differ in their environmental relevance and level of stringency. Ready biodegradability tests (e.g., OECD 301) serve as stringent screening methods requiring rapid and extensive degradation under defined conditions [20]. In contrast, inherent biodegradability tests allow for higher microbial activity and provide a more permissive assessment of degradation potential, while simulation tests offer more realistic environmental conditions that better reflect natural ecosystems [21]. Similarly, ISO methods (e.g., International Organization for Standardization (ISO 14855-1)) [22] are commonly applied to evaluate polymer biodegradation, focusing on parameters such as oxygen demand and CO_2_ evolution. Although these standardized methods ensure regulatory acceptability and comparability, their controlled conditions may not accurately reflect real-world conditions, highlighting the need for additional research conducted in more realistic environmental settings.

To mitigate chlorothalonil pollution in contaminated environments, various remediation methods have been previously employed, including electrolysis, hydrolysis, photocatalysis, phytodegradation, bioaugmentation, and biodegradation [23,24]. Among these, biodegradation is considered the most cost-effective and environmentally friendly approach and has gained significant attention for the degradation of agrochemicals, including chlorothalonil, in the environment [25]. Microorganisms utilize their enzymatic systems to break down pesticides into secondary metabolites or non-toxic end products through processes such as hydrolysis, oxidation, reduction, and co-metabolism [23]. Due to their adaptable metabolic capabilities, soil microorganisms can effectively degrade xenobiotic compounds, often using them as sole carbon and energy sources. They also interact with indigenous soil microflora and fauna, which further enhances their degradation efficiency [26,27,28].

For instance, Li et al. [29] isolated and cultured bacterial strains from three genera (*Pseudomonas*, *Enterobacter*, and *Rhodotorula*) as well as a fungal strain from the *Aspergillus* genus, from natural soil. These microorganisms successfully degraded seven types of pesticides, including chlorothalonil, chlorpyrifos, imidacloprid, carbendazim, lambda-cyhalothrin, beta-cypermethrin, and deltamethrin, at concentrations ranging from 250 to 1000 mg/L. In a different investigation, Esquirol et al. [30] isolated *Pseudomonas* sp. strain ADP from agricultural soil and demonstrated its ability to remediate s-triazine herbicides such as atrazine, ametryn, and melamine.

Various bacterial genera have been recognized because of their capacity to degrade chlorothalonil in water and soil environments. Most of these bacterial species belong to the genera *Sulfitobacter*, *Pseudoxanthomonas*, *Ochrobactrum*, *Caulobacter*, *Pseudomonas*, *Sphingobium*, *Lysobacter*, *Rhizobium*, *Bordetella*, *Marinicauda*, *Erythrobacter*, *Stakelama*, and *Oceanicaulis* [7,31,32]. However, there is an urgent need to discover new, highly effective microbial strains that can efficiently enhance the biodegradation process under laboratory and field conditions without causing secondary pollution in the ecosystem.

Many microbial strains struggle to degrade agrochemicals in open environments due to extreme environmental conditions. This limitation creates a gap between laboratory and field experiments, often leading to the failure of biodegradation processes [33]. However, various carrier materials such as sodium alginate, chitosan, polyvinyl alcohol, biochar, agar, and chitin can be used to immobilize microbes through adsorption, cross-linking, encapsulation, covalent bonding, and entrapment [34]. Immobilizing microbes with these carrier materials enhances their tolerance to toxic pollutants by providing a large surface area, strong adsorption capacity, high porosity, and improved permeability [35]. Additionally, microbial cells immobilized within carrier materials exhibit high thermal and chemical stability, enabling their reuse multiple times in liquid samples while maintaining efficient pollutant degradation [36].

In this study, the previously isolated bacterial strain *Proteus terrae* ZQ02 from polluted soil was investigated for its ability to degrade chlorothalonil in various matrices using cell encapsulation techniques. The strain was immobilized in sodium alginate microcapsules, which were optimized through orthogonal experiments to enhance stability, mechanical strength, shelf-life, and permeability. The degradation capabilities of both free and immobilized ZQ02 cells were evaluated under different stress conditions, including variations in temperature, pH, and nutrient availability. This approach enhances the strain’s field adaptability, stability, and reusability, providing a practical strategy for large-scale chlorothalonil remediation and bridging laboratory findings with field applications for sustainable pesticide management.

## 2. Materials and Methods

### 2.1. Chemicals Reagents and Mediums

Chlorothalonil technical grade with a purity of 97% was purchased from Aladdin; Chlorothalonil suspending agent with a 40% active ingredient content was purchased from Sichuan Guoguang Agrochemical Co., Ltd., Chengdu, China. and the recommended dosage was 150–175 mL/667 m^2^. Sodium alginate was purchased from Tianjin Yongda Chemical Reagent Co., Ltd., Xiqing District, Tianjin, China. Tris-HCl was purchased from Biosharp, Hefei, China. Sodium alginate (SA), dipotassium hydrogen phosphate, agar, tryptone, yeast extract, ammonium chloride, magnesium sulphate, calcium chloride (CaCl_2_) anhydrous, ferric chloride, glycerol and glutaraldehyde were purchased from Tianjin Damao Chemical Reagent Co., Ltd., Dongli District, Tianjin, China. Methanol (chromatographic grade) and acetonitrile (chromatographic grade) were purchased from Shanghai Anpu Technology Co., Ltd., Songjiang District, Shanghai, China. Methanol (analytical pure) and sodium acetate were purchased from Tianjin Baishi Chemical Co., Ltd., Yuejin Road, LiMingZhuang, Dongli District, Tianjin City, China.

Mineral salt media (MSM g/L: (NH_4_)_2_SO_4_, 2 g, MgSO_4_.7H_2_O 0.2 g, FeSO_4_.7H_2_O, 0.001 g, Na_2_HPO_4_.12H_2_O 1.5 g, KH_2_PO_4_,1.5 g). Mineral salt–carbon medium (MCM g/L: 1.0 g NaCl, 1.5 g K_2_HPO_4_, 0.5 g KH_2_PO_4_, 0.2 g MgSO_4_.7H_2_O, 1.0 g (NH_4_)_2_SO_4_, 1 g yeast extract and 1.5 g tryptone per litter of pure water, pH 7.0). Luria–Bertani medium (LB g/L: tryptone 10.00 g, yeast extract 5.00 g, NaCl 10.00 g, pH 7.0) supplemented with chlorothalonil was used for the degradation experiment. Every medium was autoclaved with steam at 121 °C for 20 min.

### 2.2. Tested Bacterial Strain, Preservation and Determination of Growth Curve

*Proteus terrae* ZQ02 was previously isolated by our research group from agriculturally contaminated soil using an enrichment culture method [37]. For long-term preservation, cryopreservation was employed. A 50% glycerol solution was prepared, autoclaved, and mixed with an equal volume of ZQ02 culture in a 1:1 ratio. The mixture was thoroughly homogenized and stored in sterilized 2 mL centrifuge tubes at −80 °C. Several studies have demonstrated that this method is effective for the long-term preservation of microbial strains.

The cryopreserved strains were inoculated at 1% into sterilized LB medium and incubated for 24 h. After incubation, the cultures were centrifuged at 5000 rpm for 4 min, and the supernatant was discarded. The cells were washed with 0.9% saline, resuspended, and adjusted to an optical density (OD) of 1.0 at 600 nm to prepare the inoculation solution. The prepared inoculum was transferred to fresh sterilized LB liquid medium at a 1% (*v*/*v*) inoculum volume and incubated at 30 °C and 160 rpm for 48 h. Each experiment was performed in triplicate. The OD at 600 nm was measured every 2 h, and a growth curve was plotted using the OD_600_ values on the y-axis and the culture time on the x-axis.

### 2.3. Chlorothalonil Degradation Conditions for Strain ZQ02

A three-way factorial design was implemented to explore the effects of initial pesticide concentration (10, 25, and 50 mg/L), temperature (25, 30, 37, and 45 °C), and pH (5, 7, and 9). The pH was adjusted using 0.1 M HCl for values ranging from 4.0 to 7.0, and 0.1 M NaOH for values between 8.0 and 9.0. Each treatment combination was replicated three times to ensure reliability. To examine the effect of temperature on the degradation process, the initial pesticide concentration was set to 50 mg/L, and pH 7.0 was maintained. Simultaneously, the effect of pH was examined at 37 °C with an initial pesticide concentration of 50 mg/L. Based on the initial findings, temperature and pH were optimized at 37 °C and pH 7.0, respectively, to evaluate the effect of different initial concentrations of chlorothalonil.

A bacterial suspension at 1.2 times optical density (OD_600_ nm), around ×10^7^ colony-forming units (CFU/mL) of ZQ02 was transferred to 100 mL of fresh sterilized LB liquid medium at a 1% inoculum volume, and chlorothalonil was added as the sole source of carbon and energy. All cultures were incubated at 150 rpm, and samples were collected after 1, 4, and 7 days. All experiments were conducted in triplicate, and three control samples were included to ensure data reliability.

For sample extraction, 5 mL of the culture was transferred to a 50 mL centrifuge tube and mixed with an equal volume of acetonitrile solvent. The mixture was vortexed for 1 min, followed by the addition of 3 g NaCl and vortexed again for 1 min. The samples were then placed in an ultrasonic bath for 25–30 min and centrifuged at 5000 rpm for 4 min. In total, 1 mL of the supernatant was extracted using a 10 mL syringe, filtered through a 0.22 µm nylon membrane filter, and analyzed for residual chlorothalonil concentration. The vials were stored at 4 °C in the dark before analysis.

The residual concentration was determined using High-Performance Liquid Chromatography (HPLC) on an Agilent 1260 system equipped with a UV detector. The chromatographic conditions were as follows: a C18 Athena reversed-phase column (4.6 × 250 mm, 5 µm, 100 Å), with the column temperature maintained at 30 °C. The injection volume was 20 µL, the flow rate was 0.8 mL/min, and the mobile phase consisted of acetonitrile/water (65:35, *v*/*v*). The absorption wavelength was set to 230 nm.

### 2.4. Fabrication of Microcapsules and Investigation of Degradation Efficiency

Sodium alginate (SA) was used as a carrier material to evaluate the degradation efficiency of strain ZQ02. A 1 mL aliquot of strain ZQ02 culture was inoculated into 5 L of LB medium (pH 7.0) and incubated at 37 °C with shaking at 150 rpm in a rotary shaker for 36 h. To obtain cell pellets, strain ZQ02 was harvested by centrifugation at 6000 rpm for 5 min. Encapsulation was carried out by the gelation method and microcapsules were easily formed by adding suspended cells in 3% sterile SA *w*/*v* in 100 mL of boiling distilled water. To create spherical immobilized microcapsules, the mixed suspension droplets were then injected dropwise using a 10 mL syringe to the well-agitated and sterilized 2% CaCl_2_ (*w*/*v*) into the solution at a height of (~10 cm). Subsequently, the microcapsules were left in the CaCl_2_ solution for 2 h at 4 °C to harden. The microcapsules were washed three times with distilled water containing 0.9% sterile sodium chloride. The washed microcapsules were drained on sterile filter paper at room temperature. After that, the dried microcapsules containing ZQ02 were sealed, put in a 50 mL sterile centrifuge tube and stored at 4 °C for further investigation. After drying, Vernier callipers were used to measure the diameter of the microcapsules.

### 2.5. Orthogonal Experimental Design for Strain ZQ02 Degradation

The concentration of SA and CaCl_2_, encapsulation time, and the number of wet pellets in the culture can influence chlorothalonil degradation at different stages. To determine the ideal carrier material ratio for the microcapsules, an orthogonal experimental design method was used, and the immobilization parameters were optimized using SPSS 26.0 (IBM SPSS Inc., Chicago, IL, USA) to generate an L9 (4^4^) orthogonal array table Table 1. In total, 0.5 g of microcapsules was used to examine degradation efficiency and treated with 50 mg/L of chlorothalonil fungicide in sterilized LB medium. The medium was incubated in a rotary shaker at 160 rpm and 30 °C for 7 days. The degradation of chlorothalonil was determined after 0, 1, 4 and 7 days. Control experiments were conducted without cells under the same conditions. There were three replicates for each treatment and control. To optimize the encapsulation conditions, the chlorothalonil degradation rate constant and *DT*_50_ were used as indicators [38].

### 2.6. Scanning Electron Microscope (SEM) Observation of Microcapsules

Fresh microcapsules with and without ZQ02 biomass were prepared for the observation and analysis by scanning electron microscopy (SEM). The morphology of microcapsules and the distribution of bacteria were observed before and after encapsulation of wet cells. The SEM samples were prepared by the critical point drying method, the procedure was as follows.

After preparing the microcapsules containing bacterial cells and the microcapsules without free ZQ02 cells, both samples were fixed for over four hours with 2.5% glutaraldehyde in phosphate buffer (pH 7.0) and then rinsed three times in the phosphate buffer. They were then postfixed with 1% osmium tetroxide (OsO_4_) in phosphate buffer (pH 7.0) for an hour and then washed three times in the phosphate buffer. For dehydration, the specimens were treated with a graded series of ethanol (50%, 70%, 80%, 90%, 95% and 100%) for about 15 to 20 min at each step. In the end, the specimens were dehydrated in a Leica Model CDP 300 critical point dryer (Leica Microsystems GmbH, Wetzlar, Germany) using liquid CO_2_. Finally, for coating and observation the dehydrated specimens were coated with platinum-17 palladium in a Leica Model ACE 600 ion sputter (Leica Microsystems GmbH, Wetzlar, Germany) and observed using a Thermo Fisher Scientific Verios 460 FESEM (Thermo Fisher Scientific Inc., Waltham, MA, USA).

### 2.7. Characteristics of Microcapsules

#### 2.7.1. Determination of Mass Transfer Performance of Microcapsules

Five microsphere preparations containing sodium alginate, wet cells of the degrading bacteria, and calcium chloride—which were similar in shape and size—were taken. All the microspheres were placed in centrifuge tubes and were mixed with 5 mL of red ink solution. After 1 min they were removed and washed three times with 0.9% normal saline to observe the discoloration of the microcapsules. The degree of discoloration was used to indirectly indicate the mass transfer ability of microspheres containing degrading bacteria. The more “+” the darker the colour of the microcapsules, indicating stronger mass transfer ability.

#### 2.7.2. Determination of Mechanical Strength and Equilibrium Volume Content (EWC)

The mechanical strength of microcapsules was investigated using the method proposed by Zheng et al. [39] with some modifications. A total of 100 microcapsules in a 250 mL flask were incubated at 37 °C at 250 rpm. The broken and intact microcapsules were counted, and their mechanical strength was examined.

The EWC of microcapsules was examined according to the method of Zhang et al., [40] with possible adjustments. Five microcapsules were placed in an incubator at 20 °C and 65% of relative humidity for 48 h. After that, all the microcapsules were dried at 60 °C for 2 h. The mass of the microcapsules was measured before and after drying by analytical balance. Finally, the equilibrium volume content was measured by formula.

#### 2.7.3. Reusability of Microcapsules

In a 100 mL sterilized shake flask with LB medium containing 50 mg/L of chlorothalonil with a pH of 7, 3.0 g of microcapsules were added and incubated in a rotary shaker at 160 rpm and temperature of 37 °C for 7 days. The degradation of chlorothalonil was determined after 0, 1, and 3 days. After that, all microcapsules were washed with distilled water containing 0.9% saline solution, and the same protocol was repeated. There were three replicates for each treatment and control.

#### 2.7.4. Determination of Permeability of Microcapsules

After adding 4 g of microcapsules to a 100 mL flask containing 20 mL of methylene blue solution (OD_406_ nm), the mixture was incubated at 37 °C to measure the permeability. The control was performed under identical conditions without ZQ02 microcapsules. A UV-1800 spectrometer was used to measure the solution’s OD_406_ after 24 h.

#### 2.7.5. Determination of Size and Suitable Dose of Microcapsules

The diameter of the microspheres was measured using a digital Vernier calliper. A total of 100 well-degraded bacterial preparations were selected, and the size of each bead was measured. The various doses of microcapsules (0.5, 1.0, 3.0 and 5.0 g) were taken and treated with 50 mg/L chlorothalonil. The degradation of fungicide was determined in a temperature-controlled rotary shaker at 160 rpm and at 30 °C for 7 days.

#### 2.7.6. Determination of Storage Life of Microcapsules

For storage stability evaluation, microcapsules were stored at 4 °C for 5 weeks after preparation. For biodegradation assay microcapsules were added into a 100 mL flask with 50 mL LB medium and 50 mg/L chlorothalonil at pH 7 and incubated at 37 °C for 24 h. After 24 h, the residual concentration was analyzed by HPLC.

### 2.8. Degradation of Chlorothalonil-Contaminated Soil Using Microcapsules

The soil from the (0–20 cm) surface layer was collected from the vegetable field located in the experimental station of SCAU, Guangzhou, P.R China. After that, it was kept in polyethylene bags and bought to the laboratory, air-dried and sieved through a 2 mm mesh to remove stones, debris and above-ground plant materials. The soil was sterilized to kill the microbial species at 121 °C for 2 h. A total of 5 kg of soil was placed in the plastic container with dimensions of 40 cm × 22 cm × 71 cm, and treated with the concentration of (10, 20 and 30 mg/L) with 5 g of ZQ02 microcapsules. The control was performed with microcapsules lacking ZQ02 cells. The water content was adjusted to 60% of the maximum water-holding capacity of the soil by the addition of sterile water. Approximately 50 g of soil sample was randomly collected after 0, 1, 7, 14, 21, 28 and 35 days. The experiment was performed as triplicate.

### 2.9. Degradation of Chlorothalonil in Tomato Vegetated Soil

The tomato vegetable seedlings were bought from Guangzhou vegetable market, soaked in distilled water for a while and planted in pots. The dimensions of plastic container were 40 cm × 22 cm × 71 cm, containing 9 kg natural and brown soil in a 2:1 ratio. The pots were divided into four groups: control, blank, treatment with ZQ02 microcapsules and free cells. In the treatment groups, 5 g microcapsules per 1 kg soil and 5 mL bacterial solution per 1 kg soil were added. In each pot, 20 tomato plants were planted and spiked with chlorothalonil at a concentration of 20 mg/L. The rhizosphere soil samples were collected from at least five different locations per plot, and we mixed them thoroughly to obtain a homogenous sample on day 0, 1, 7, 14, 21 and 28. For the determination of fungicide residues, plant tissue (roots and shoots) samples were collected. The evaluation of residues in plant matrix was measured by using a rotary evaporator. We took 5 g of the plant sample and placed it in a 50 mL centrifuge tube, added 10 mL acetonitrile and swirled it for 1 min. The centrifuge tube with the sample was placed in the ultrasonic cleaner for 20 min and 3 g NaCl was added afterward. The centrifuge tubes were centrifuged at 4000 rpm for 5 min and 5 mL of the organic solvent was transferred to the pear-shaped bottle in a 45 °C rotary evaporator, and the concentrate was dried. After that, the concentrate was eluted and redissolved in 1 mL of acetonitrile from a pear-shaped flask and chlorophyll content was removed by adding 1 mL of green supernatant in a 2 mL tube containing 0.1 g primary secondary amine (PSA) and 0.1 g graphitized carbon black (GCB). The tubes were centrifuged at 100 rpm for 2 min, the supernatant was sucked by a disposable syringe and passed through 0.22 µm microporous organic membrane filter, and it was collected in a brown HPLC vial for detection.

### 2.10. Statistical Analysis

In this paper, the mean, standard error, linear regression and so on were measured with Excel 2013 software processing. Tables and so on were drawn with GraphPad prism 5 and Excel 2013 software processing. The arithmetic averages, standard deviations and so on were determined via software Excel 2013 (Redmound, WA, USA). Statistically significant variances were evaluated by using a one-way ANOVA with SPSS 26.0 (IBM SPSS Inc., Chicago, IL, USA) and statistical significance was set at *p* < 0.05.

The degradation rate is calculated as follows:

$$\mathrm{Degradation}\;\%=\frac{c_{0}-c_{f}}{c_{0}}\times 100$$

where *C*_0_ and *C_f_* is the initial and final concentration (mg/kg) of residues before and after treatment, respectively.

The degradation kinetics accords with the first-order kinetic model to explain the degradation efficiency by the following formula:

$$c=c_{0}e^{-kt}$$

where *C* was the chlorothalonil concentration (mg/L) at the time of *t* (*d*), *C*_0_ was the initial agrochemical dosage (mg/L), and *k* was the kinetic constant.

The half-life of chlorothalonil residues in tomato vegetated soil was calculated by half-life *T* = *ln* 2*k*^−1^ (d).

## 3. Results and Discussion

### 3.1. Growth Curve of Chlorothalonil-Degrading Strain

After 48 h of incubation and detection of OD_600_ of strain ZQ02, the growth curve of degrading bacterium was obtained as shown in Figure S1. In a nutrient medium, a bacterial strain ZQ02 produced beige, glossy, wet colonies that smelled like ammonia. At the delay period of every 2 h, bacteria adapted to a new environment and reserved sufficient enzymes and energy for cell volume increase, rapid metabolic activity and cell proliferation; subsequently, from 6 to 24 h, the logarithmic growth increased significantly in a geometric order. The lag phase was 8 to 36 h, and during this period nutrients in the medium were consumed and the metabolic activity was increased rapidly. After 36 h the rate of microbial growth began to decline gradually. The rate of bacterial autolysis and death was faster than that of reproduction which reflected the trend of decline after 40 h due to limited availability of nutrients, oxygen and adaptation to the new environment. However, our results are in line with earlier research conducted by Yang et al. [41] and Li et al. [29]; they found that the maximum growth of *B. subtilis* (BY-2) and *B. velezensis* (M2) was achieved after 20 to 39 h of incubation, respectively.

A bacterial strain, *Bacillus brevis*, was studied for phenol degradation, and its exponential growth was investigated. The study reported that bacterial growth declined due to reduced oxygen levels and substrate availability within the biomass, leading to a decrease in pH and the onset of cell autolysis [42]. In another experiment, the growth patterns of various bacterial strains were examined during cellulose degradation. The results revealed that after 40 h of incubation, bacterial growth declined rapidly due to nutrient depletion [43]. Previous studies have employed a growth inhibition test using activated sludge bacteria, in accordance with ISO 15522 [44], to evaluate the potential toxicity of test compounds on the growth of mixed aerobic bacterial populations present in activated sludge. The inhibitory effect is limited to bacteria capable of growing on the selected organic test medium. The standard defines the required equipment, reagents, test conditions, and experimental procedures. The principle of the method is to expose mixed activated sludge bacteria in the early exponential growth phase to varying concentrations of a test chemical. Subsequently, microbial growth is monitored by measuring turbidity at 530 nm. This method has been validated using reference substances such as phenol, various chlorophenols, and 2,4-dinitrophenol [36].

### 3.2. Characterization of the Strain ZQ02 for the Biodegradation of Chlorothalonil

**Figure 1:**
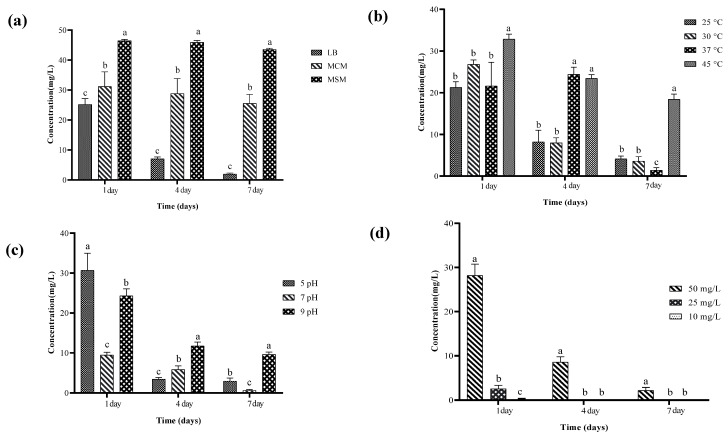
(**a**) Ability of strain ZQ02 to degrade chlorothalonil in various media. (**b**) Effect of temperature on activation of ZQ02 free cells and degradation of chlorothalonil. (**c**) Effect of pH on activation of free cells and degradation of chlorothalonil. (**d**) Effect of various concentrations on activation of free cells and degradation of chlorothalonil. The data was analyzed using Duncan’s tests, and the findings are shown as the mean ± standard deviation. While the existence of a similar letter suggests that the strains are not substantially different (*p* < 0.05), the presence of distinct letters on the bars shows a significant variation across different days.

The biodegradation kinetics of chlorothalonil were evaluated in 100 mL media types (LB, MSM and MCM) with 50 mg/L of chlorothalonil fungicide for 7 days. Findings revealed that the concentration of chlorothalonil was decreased rapidly in the treatment group inoculated with the degrading bacterium ZQ02 as shown in Figure 1a. Hydrolysis also performs a critical role in the liquid medium degradation; after 7 days, 10% of chlorothalonil was hydrolysed in the uninoculated control group. In the treatment group, the amount of chlorothalonil decreased significantly relative to beginning and almost half of chlorothalonil was degraded in the first day. After 7 days of culture, 95.57% of chlorothalonil was effectively removed. This experiment concluded that bacterial strain ZQ02 has the ability to degrade chlorothalonil efficiently and could be considered a superior candidate for the clean-up of the environment. In MSM media, after 7 days of incubation there was no degradation of chlorothalonil fungicide observed. Meanwhile, as compared with MCM media, free cells of strain ZQ02 were slightly active due to the presence of nutrients in MCM media and degraded 41.54% of chlorothalonil fungicide with an initial concentration of 50 mg/L. This study determined that bacterial strain exhibits degradation ability and performed efficiently in LB media, showing slow degradation activity in MCM media. Due to the absence of nutrients in MCM medium, the growth and degradation of bacterial strain were not satisfactory.

After seven days at a temperature of 37 °C and neutral pH, the growth of strain ZQ02 was at its maximum, and it degraded a high amount of chlorothalonil with the concentration of 50 mg/L (98.66% and 97.13%), respectively, as shown in Figure 1b,c. Meanwhile, at a temperature range between 20 °C and 45 °C the degradation of chlorothalonil was low due to less growth of ZQ02. With the concentration of (10, 25 and 50 mg/L) of chlorothalonil, ZQ02 degraded (94.24, 97.01 and 95.01%) at day 1, 4 and 7, respectively, as shown in Figure 1d. It concluded that strain ZQ02 could degrade chlorothalonil residues in a wide range of temperatures ranging from 20 to 45 °C and that the optimal temperature was 37 °C; meanwhile, it shows greater degradation ability at pH value 7 and lost its degradation potential when the pH value was less than 5. When the pH value was 9, chlorothalonil was chemically hydrolysed because it was unstable in alkaline environments.

This study compares the newly isolated chlorothalonil-degrading bacterium ZQ02 with previously reported strains involved in chlorothalonil remediation. A bacterial strain, *Stenotrophomonas* sp. H4, was screened from agricultural soil and evaluated for its degradation efficiency. The results revealed that this strain degraded 82.2% of chlorothalonil at an initial concentration of 20 mg/L, pH 7.0, and 30 °C after 7 days in liquid culture [45]. Similarly, *Enterobacter cloacae* was isolated and found to degrade 97.4% of chlorothalonil at a concentration of 20 mg/L, pH 7.0, and a temperature range of 30–35 °C after 48 h of incubation in an aqueous solution [6].

In another study, an indigenous bacterial strain, *Pseudomonas* sp. CDS-8, was tested for its degradation efficiency. The study concluded that in combination with titanium oxide nanoparticles, 90.73% of chlorothalonil removal was achieved at an initial concentration of 50 mg/L, pH 7, and 30 °C in MCM medium [46]. Similarly, *Pseudomonas* sp. CTN-4 was reported to degrade 96% of chlorothalonil under the identical conditions [47].

Additionally, *Stenotrophomonas acidaminiphila* BJ1 utilized chlorothalonil as its sole carbon source and demonstrated degradation efficiencies of 91.5%, 89.4%, 86.5%, and 83.5% at initial concentrations of 50, 100, 200, and 300 mg/L, respectively, after 96 h of incubation under optimal conditions (30 °C, pH 7.0) [48].

### 3.3. Fabrication of Microcapsules and Suitable Dose for Degradation of Chlorothalonil

**Figure 2:**
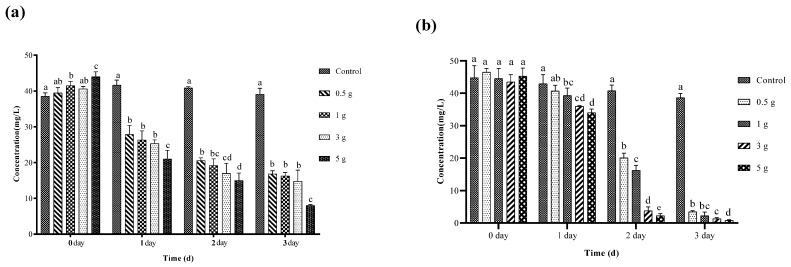
(**a**) Determination of suitable dose of microcapsules in MCM media. In MCM media the effective dose of microcapsules was 5 g. (**b**) Determination of suitable dose of microcapsules in LB media. In LB media all dosages perform efficiently due to availability of high nutrients in LB media. The data was analyzed using Duncan’s tests, and the findings are shown as the mean ± standard deviation. While the existence of a similar letter suggests that the strains are not substantially different (*p* < 0.05), the presence of distinct letters on the bars shows a significant variation across different days.

The wet cell pellets were obtained by centrifugation and mixed with the SA solution. The results showed that CaCl_2_ acted as a chemical cross-linking agent for SA and the wet pellets of *Proteus terrae* ZQ02. After washing with distilled water, pale yellowish-white microcapsules were obtained, whereas microcapsules lacking free cells appeared white, as shown in Figure S2. To determine the optimal dosages of microcapsules for chlorothalonil degradation, experiments were conducted in LB and MCM media types, as illustrated in Figure 2. The degradation rate of chlorothalonil increased significantly with higher dosages of microcapsules compared to lower dosages. However, only a slight difference in biodegradation efficiency was observed between 3 g and 5 g of microcapsules after 3 days. Based on these findings, this study concludes that 3 g of microcapsules is the optimal dosage for efficient chlorothalonil degradation.

Furthermore, this study highlights that carrier materials act as efficient catalysts, accelerating the degradation of chlorothalonil fungicide, particularly in nutrient-limited media.

Recently, Fang et al. [49] employed an immobilization strategy to enhance the degradation efficiency of the chlorpyrifos-degrading strain *Cupriavidus nantongensis* X1T by immobilizing it onto four carrier materials: SA, diatomite, chitosan, and polyvinyl alcohol. The study concluded that SA and chitosan were the most effective immobilization agents, achieving a 96.6% chlorpyrifos degradation at an initial concentration of 20 mg/L within 24 h in an aqueous solution.

Similarly, another study investigated chlorpyrifos degradation using SA microcapsules. The findings revealed that 2.5 g of microcapsules efficiently degraded 99.8% of chlorpyrifos at an initial concentration of 100 mg/L [50]. In a separate study, calcium alginate-immobilized *Phanerochaete chrysosporium* beads were tested at different dosages (1, 2, 3, and 4 g) to assess their effectiveness in bisphenol removal. The results confirmed that degradation proficiency increased with higher biomass dosage. In contrast, the 1 g dosage group achieved 76% bisphenol removal; all other groups reached 100% degradation within 48 h [51]. Additionally, the degradation of phenol was studied using varying dosages (0.1 to 5 g/L) of immobilized *Chlorophyta* species. The results indicated that a 5 g/L dosage was the most effective, achieving 95.3% phenol removal [52].

### 3.4. Orthogonal Array Configuration Experiment

The concentration of carrier materials such as SA and CaCl_2_ can increase or decrease the degradation potential of microcapsules, stability, permeability and mechanical strength [53,54]. To evaluate the optimal degradation efficiency of microcapsules, stability, reusability, permeability and long shelf-life an orthogonal experiment was designed.

**Figure 3:**
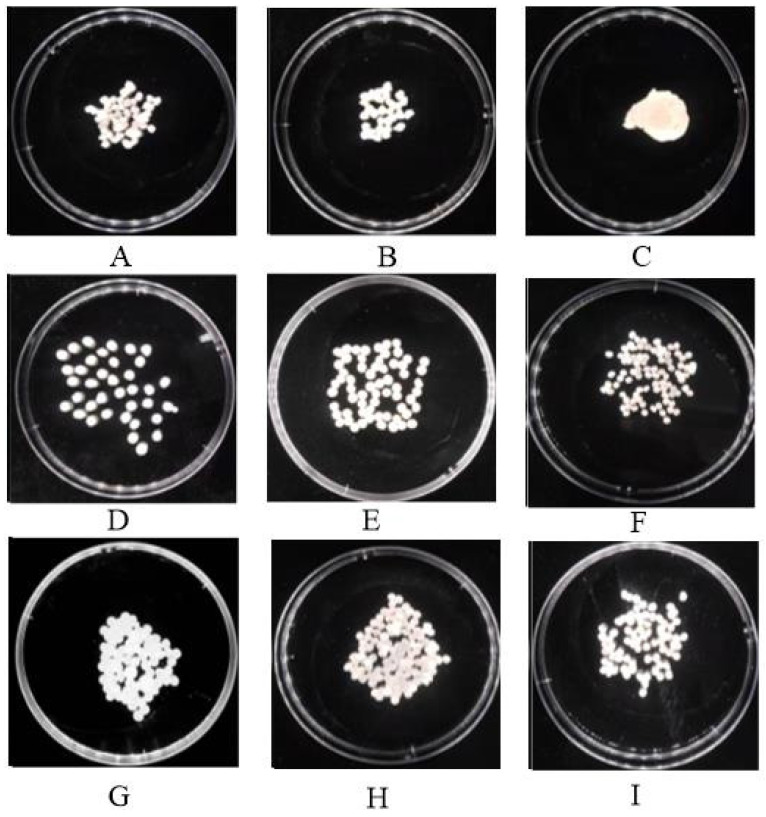
Different letters show various concentrations of carrier’s materials for microcapsule formation and their structural size and properties. (**A**) denote 3% SA, 60 g/L ZQ02 wet cells and 3% CaCl_2_ (microcapsules were not in standard size), (**B**) exhibit 2% SA, 40 g/L ZQ02 wet cells and 3% CaCl_2_ (microcapsules make clusters), (**C**) represent 1% SA, 20 g/L ZQ02 wet cells and 3% CaCl_2_ (microcapsules were fragile and irregular shaped), (**D**) illustrate 3% SA, 60 g/L ZQ02 wet cells and 2% CaCl_2_ (microcapsules were good in stability and mechanical strength with standard size), (**E**) indicate 2% SA, 40 g/L ZQ02 wet cells and 2% CaCl_2_ (microcapsules were in standard size but stability and mechanical strength was not good for long time storage), (**F**) reveal 1% SA, 20 g/L ZQ02 wet cells and 2% CaCl_2_ (all the microcapsules formation by this formula were smaller and easily broken), (**G**) appear 3% SA, 60 g/L ZQ02 wet cells and 1% CaCl_2_ (all the microcapsules were intact each other and make clusters), (**H**) display 2% SA, 40 g/L ZQ02 wet cells and 1% CaCl_2_ (smaller in size and easy to broken), and finally (**I**) reveal 1% SA, 20 g/L ZQ02 wet cells and 1% CaCl_2_ (fragile microcapsules with irregular shaped).

The effects of 1–3% SA solution on the encapsulation performance and mechanical strength of the microcapsules are presented in Table 1. Figure 3 represents the physical appearance of microcapsules with different concentrations of SA. Results revealed that when the concentration of sodium alginate was 1%, the microcapsules were rigid, tired and difficult to pelletize. At a 2% concentration of sodium alginate the mass transfer property of the microcapsules was optimal, but due to the less mechanical strength the shelf-life decreased gradually, and they were easy to break. At a 3% concentration of sodium alginate the granulation of microcapsules was relatively easy and the mechanical strength, mass transfer ability, permeability and stability were better. Thus, this study concluded that 3% of sodium alginate was the best concentration for the encapsulation of strain ZQ02.

The concentration of CaCl_2_ had significant effects on the degradation performance and properties of microcapsules. Results in Table 1 and Figure 3 show that the 2% and 3% concentration were much superior in cross-linking than the 1% concentration, while no significant differences was observed between the concentrations of 2% and 3%. Lower concentration of CaCl_2_ results in fragile, soft and smaller microcapsules which are easily broken and unable to be reused. The higher concentration of CaCl_2_ affects stability, mechanical strength and the degradation performance of microcapsules of chlorothalonil residues. However, in comparison with the SA concentration, the concentration of CaCl_2_ plays a lesser role in the formation of microcapsules because of the gelation process. Further results showed that a 2% concentration of CaCl_2_ is suitable for the cross-linking mechanism and balances the mass transfer of microcapsules.

The effect of ZQ02 wet cells at concentrations of 2–6% was studied for the degradation of chlorothalonil fungicide residues and to observe the properties of microcapsules. Using a high concentration of wet cells of ZQ02 changed the colour of microcapsules to dense yellow as compared to lower concentrations. Figure 3 represents the shapes and colours of microcapsules using different concentrations of wet cells, SA and CaCl_2_ at various encapsulation times. The concentration of 6 g of wet cells was found to be optimal and significantly increased the degradation rate as compared to lower and higher concentrations of wet cells. At a higher concentration a large number of microcapsules were broken which gradually decreased the degradation efficiency of microcapsules due to a reduction in porosity and surface area because the existence of more wet cells covered more pore spaces, resulting in reduced mechanical strength. However, the optimal conditions for microcapsules were observed with 60 g/L ZQ02 wet cells, 3% SA and 2% CaCl_2_ in 100 mL to degrade a high amount of chlorothalonil fungicide.

Encapsulation time plays an essential role in the cross-linking of degrading bacteria with carrier materials. Results demonstrated that encapsulation times ranging from 15 min to 12 h efficiently degraded the concentration of chlorothalonil as compared to longer encapsulation times. Moreover, this study concluded that the formation of higher encapsulation time was unbaled to recycle and easily broken.

To find the ideal carrier material concentration and encapsulation duration, several formulations were designed for the degradation of prometryn and other s-triazine herbicides. The findings showed that 2% and 3% CaCl_2_ were more effective than 1% CaCl_2_, though no substantial difference was observed between 2% and 3%. Similarly, 10% and 12% polyvinyl alcohol were found to be more suitable for degradation, but no significant difference was noted compared to 8% polyvinyl alcohol. To determine the best encapsulation time, bacterial entrapment was conducted for up to 36 h, and results indicated that 24 and 36 h were superior; however, no significant difference was observed compared to 12 h [55]. A recent study was conducted to optimize the immobilization conditions of bacteria for degrading multiple pesticides. The findings revealed that a formulation comprising 1 g of wet bacterial cells, 1% SA, and 3% CaCl_2_, with an optimized immobilization time, degraded lactofen, acetamiprid, and carbendazim more efficiently than other formulations [56]. In another study, the degradation of chlorpyrifos was investigated using *Bacillus* sp. laccase immobilized with iron magnetic nanoparticles. The results revealed that the highest laccase activity recovery was achieved with a laccase concentration of 75 mg/mL, 200 mg/mL magnetic nanoparticles, 0.3% cross-linking with carbodiimide, and a 3 h cross-linking time [57].

### 3.5. Scanning Electron Microscope (SEM) Observation of Microcapsules

**Figure 4:**
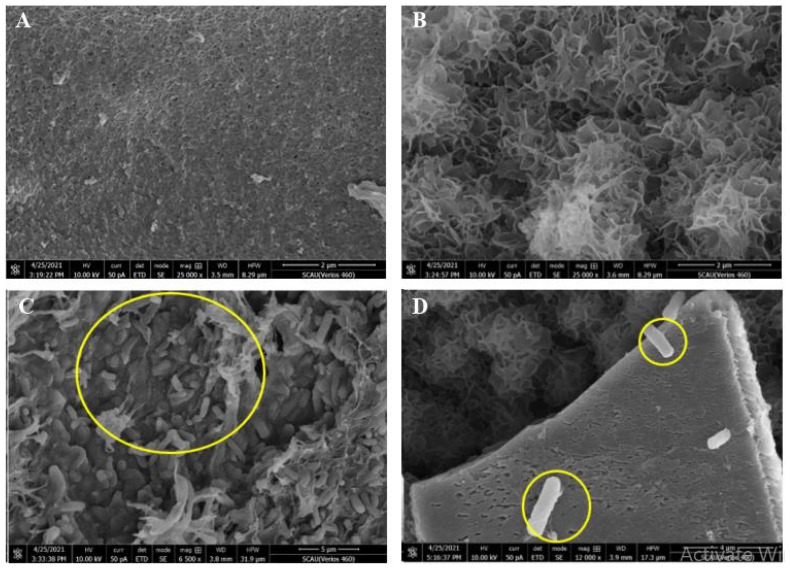
(**A**) Inside structure of microcapsules lacking ZQ02 wet cells (structure on the surface 25,000× with scale bar 2 µm). (**B**) Outside structure of microcapsules lacking ZQ02 free cells (structure on the surface 25,000× with scale bar 2 µm). (**C**) Inside structure of microcapsules with ZQ02 wet cells (structure on the surface 6500× with scale bar 5 µm). (**D**) Outside structure of microcapsules with ZQ02 wet cells (structure on the surface 12,000× with scale bar 4 µm).

The microcapsules with and without wet cells of ZQ02 and microcapsules lacking wet cells of ZQ02 were examined using SEM. The internal and external structures of microcapsules lacking free cells of ZQ02 are shown in Figure 4A,B. The outside structure of microcapsules presented a flat structure due to absence of free cells of ZQ02. Meanwhile, the inside structure Figure 4C of microcapsules represented that it comprised a large number of free spaces and looked like a honeycomb structure, which provided enough surface area for free cells of ZQ02. Additionally, the inside structure of microcapsules showed a finger-like instead of flat structure which could be attributed to rod shaped bacteria with flagella existence in the microcapsules. Porous surfaces on inner and outer structures were easily visible in both structures. These pores act as channels for the transportation of ZQ02 free cells in microcapsules and are convenient for encapsulation as well as for cross-linking.

Further images showed that free bacterial cells attached to the carrier materials and formed stable encapsulation. Even though SA is frequently employed as a support material in industrial research, it improves surface characteristics including diffusion and porosity to improve interactions [58]. This structural stability of ZQ02 indicates unique adaptability to the harmful effects of chlorothalonil, pointing to efficient cellular processes such as complex cell wall architecture or the existence of efflux pumps for chlorothalonil tolerance or detoxification. These characteristics are critical for survival in contaminated environments and could improve the isolate’s effectiveness in pollutant-targeting bioremediation. Additionally, Figure 4D demonstrates that the microsphere’s surface perforations did not completely fill in, leaving some open areas that might permit the substrate to enter the microsphere [59,60]. However, chlorothalonil breakdown could be accelerated by such a structure because it increases the specific surface area and carrier porosity, which lowers diffusion resistance and makes it easier for nutrients and oxygen to be transported.

This study concluded that the structure of sodium alginate microspheres did not change significantly before and after fixation. The bacteria were widely distributed in SA microspheres, and the shape and structure of degrading bacterial strain were normal, which indicates that the strain could adhere to the carrier materials and form stable immobilized bacterial degradation complex. Hence, the alginate as a carrier material can be considered to be a suitable choice for the encapsulation of ZQ02 bacteria for the removal of chlorothalonil residues in various environments.

### 3.6. Characteristics of Microcapsules and Their Reusability

**Figure 5:**
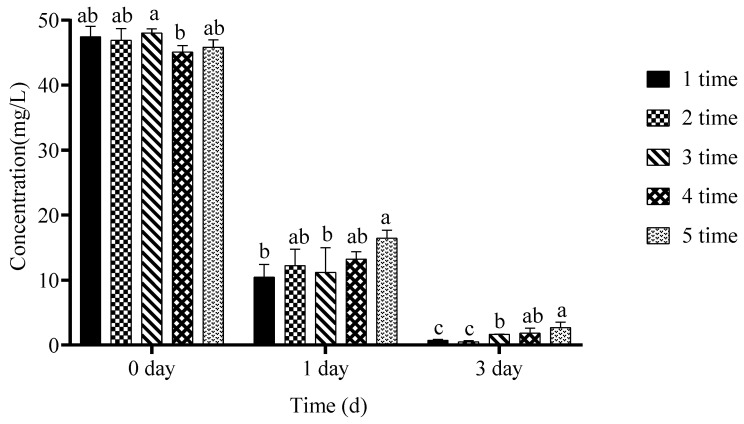
Reusability of microcapsules. The data was analyzed using Duncan’s tests, and the findings are shown as the mean ± standard deviation. While the existence of a similar letter suggests that the strains are not substantially different (*p* < 0.05), the presence of distinct letters on the bars shows a significant variation across different days.

The reusability of microcapsules is exhibited in Figure 5. The removal percentage of chlorothalonil after five recycles showed that reuse of microcapsules significantly degraded chlorothalonil in a feasible manner, and the results concluded that 3 g was the suitable dosage for the chlorothalonil breakdown. The degradation rate of chlorothalonil was 98.86% after the first cycle. The degradation rate was 98.47% after the second cycle. The degradation rate decreased slightly with the repeated reuse of microcapsules. After third, fourth and fifth cycles, the degradation rates were 96.52%, 95.90% and 94.10% respectively after 3 days of incubation in LB medium, which indicating that the biodegradable bacteria preparation could be reused for more than five cycles. It also showed that the stability and degradation performance of microcapsules after repeated use were satisfactory. An immobilized bacterial strain WL08 was able to achieve 84.16% of dimethomorph removal efficiently following the tenth cycle, indicating that WL08 immobilized on bamboo charcoal (BC) with SA had improved reusability. This was mainly because BC, a nutrient carrier with numerous porosities, could offer sufficient room for bacteria to flourish [32].

The mass transfer of microcapsules with and without degrading bacteria was investigated using methylene blue solution. Results indicated that after 1 min of treatment, the colour of microcapsules with bacterial cells was darker compared to microcapsules lacking cells of degrading bacteria. This study concluded that the darker colour of microcapsules indicated great mass transfer in the formation of microcapsules.

Microcapsules with and without ZQ02 wet cells had a spherical shape, and a large number of randomly distributed internal cavities of different sizes were present. The results of this analysis showed that strain ZQ02 preferred growing at the edges of the microcapsules. Bacterial cells were found in each of the bacterial clusters (colonies) that were substantially more numerous in this location. Over 90% of the microcapsules ranged from 2.9 and 3.1 mm in size. Microcapsules with an average size of 3 mm were successful in the chlorothalonil degradation process.

The OD_406_ values of microcapsules and control were measured at 0 h by a UV spectrophotometer, and the results indicated that their values were 0.024 and 0.028 respectively. After 24 h of incubation the OD value of microcapsules was increased to 1.13, which was greater than control microcapsules 0.076. However, this study concluded that microcapsules were more permeable and play a crucial role in the biodegradation of chlorothalonil. Cell immobilization may help stabilize membrane permeability and shield against the harmful effects of excessive substrate concentration, which would accelerate the degradation rate [61,62].

**Figure 6:**
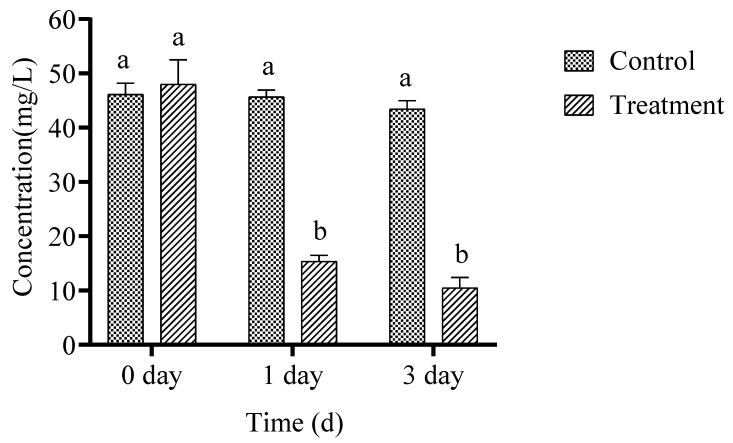
Shelf-life of microcapsules. The data was analyzed using Duncan’s tests, and the findings are shown as the mean ± standard deviation. While the existence of a similar letter suggests that the strains are not substantially different (*p* < 0.05), the presence of distinct letters on the bars shows a significant variation across different days.

The elasticity and stability of microcapsules was satisfactory as observed in reusability experiments, and the elasticity of microcapsules was enhanced by increasing the concentration of SA. This finding was observed by an orthogonal array design experiment. Microcapsules were observed after high-speed rotation and all the microcapsules were in their original position with no beads broken, which showed that the mechanical strength of microcapsules was good. The EWC of microcapsules after 48 h of drying was 142.30%. Compared with one type of carrier material, the combination of two or more types of materials significantly enhanced the stability, permeability, mechanical strength and degradation ability of immobilized cells [32]. After 5 weeks of storage the shelf-life of encapsulated beads remained stable and performed with the same efficiency for chlorothalonil breakdown. The findings on the shelf-life encapsulated beads revealed that they had the potential to degrade 78.05% fungicide with an initial concentration of 50 mg/L in LB media Figure 6. This study concluded that this formulation could be stored long-term at a temperature of 4 °C. The storage stability of BC-SA beads was investigated for up to 150 days. The results showed that the immobilized beads effectively degraded dimethomorph, achieving 91.39% degradation on day 60 and 82.20% on day 150 [40].

### 3.7. Degradation of Chlorothalonil-Contaminated Soil Using Microcapsules

**Figure 7:**
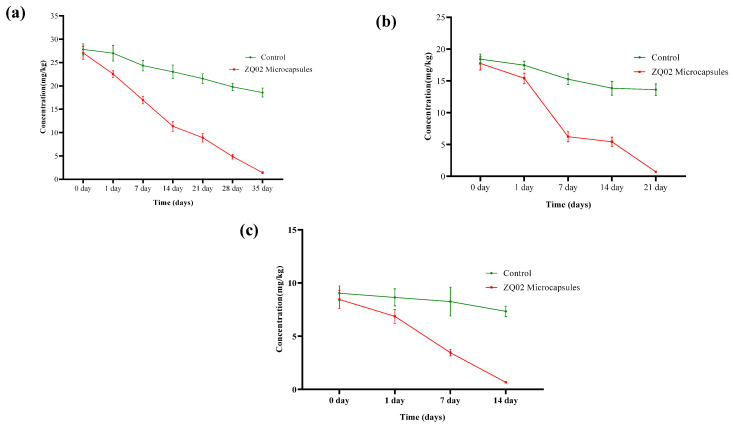
(**a**) Biodegradation of chlorothalonil in soil at 30 mg/kg. (**b**) Biodegradation of chlorothalonil in soil at 20 mg/kg. (**c**) Biodegradation of chlorothalonil in soil at 10 mg/kg.

Numerous test techniques based on various concepts have been developed as a result of the diverse spectrum of biodegradation processes that occur in both natural environments and technical systems for wastewater and solid waste treatment. Along with well-known quality assurance frameworks like GLP, EN 45000, and ISO 9000, [63,64,65] standardized methods like those created by the Organization for Economic Co-operation and Development and the International Organization for Standardization are widely used to guarantee that test results are accepted globally by regulatory bodies and stakeholders [66]. The imprudent use of chlorothalonil and its high affinity for soil particles leads to soil pollution and poses a severe threat to non-target organisms [67]. The initial concentrations of chlorothalonil in the soil treatments were 30, 20, and 10 mg/L, respectively Figure 7. In the control group, chlorothalonil degradation occurred slowly, with degradation rates of 33.17%, 26.07%, and 18.89%, respectively. In contrast, the application of microcapsules significantly enhanced the degradation of chlorothalonil in soil, demonstrating an effective remediation potential for contaminated environments. In the treated pots, degradation efficiencies reached 94.73%, 96.02%, and 92.17% at 14, 21, and 35 days, respectively. These results confirm that chlorothalonil residues can be efficiently removed through the application of ZQ02-immobilized microcapsules. The strong degradation capability of strain ZQ02 supports its potential for large-scale application in the remediation of chlorothalonil-contaminated agricultural fields.

The kinetic parameters, including the degradation model, half-life, rate constants, and coefficient of determination (R^2^), are presented in Table 2. The results indicated that chlorothalonil degradation in soil followed a first-order kinetic model, with correlation coefficients ranging from 0.92 to 0.99. The degradation rate constants (k) in the control group were 0.013, 0.021, and 0.029 day^−1^, respectively, while those in the treatment group increased markedly to 0.127, 0.227, and 0.373 day^−1^, respectively. Correspondingly, the half-lives of chlorothalonil in the treated soils at 30, 20, and 10 mg/L were significantly reduced to 5.45, 3.05, and 1.85 days, respectively. The primary external factor influencing degradation in sterilized soil was photolysis. An increase in pesticide concentration results in the formation of a thicker molecular layer per unit area, reducing the amount of light energy received per molecule and ultimately prolonging bond cleavage time under constant light intensity [68].

The degradation of chlorothalonil was investigated through the bioaugmentation of bacterial strains *Pseudochrobactrum* sp. BSQ1 and *Massilia* sp. BLM18 at two different concentrations (50 mg/kg and 100 mg/kg, respectively). The results revealed that both bacterial strains completely degraded chlorothalonil within 35 days, with half-lives of 6.8 days for strain BSQ1 and 9.8 days for strain BLM18. However, at the higher fungicide concentration (100 mg/kg), bioaugmentation with strains BSQ1 and BLM18 reduced chlorothalonil by 76.7% and 62.0%, respectively, within the same period [26].

In another study, eight native bacterial strains suitable for bioaugmentation were isolated from banana-cultivated soil. These microorganisms demonstrated the ability to tolerate and degrade chlorothalonil. The results showed that at a spiked concentration of 2000 ng/g, chlorothalonil degradation under bioaugmentation conditions reached 100% within 21 days, whereas under natural attenuation conditions, degradation reached 98.5% after 35 days [8]. The degradation of several pesticides, such as lactofen, acetamiprid, and carbendazim, was examined in soil and water. This was accomplished using bacterial agents immobilized in (SA) that contained the strains of *Bacillus* sp. Za, *Pigmentiphaga* sp. D-2, and *Rhodococcus* sp. DJL-6 that degrade lactofen, acetamiprid, and carbendazim, respectively.

The study revealed that, compared to free bacterial cells, the immobilized bacterial agents efficiently degraded 92.50% and 98.50% of lactofen, 91.05% and 99.89% of acetamiprid, and 88.43% and 98.99% of carbendazim within 21 days in soil and 7 days in water, respectively [56]. Another study examined the biodegradation of cypermethrin by (SA) as carrier material for immobilization of bacterial co-culture (*B. thuringiensis* strain SG4 and *Bacillus* sp. strain SG2). On days 5 and 10, the degradation efficiency was 30.0% and 75.0%, respectively, while the maximum degradation of 87.0% was observed on day 15. In contrast, on days 5, 10, and 15, the rates of cypermethrin degradation utilizing immobilized agar discs were 34.0%, 74.2%, and 91.3%, respectively [69]. Utilizing *Leucobacter* sp. JW-1 bacteria immobilized on polyvinyl alcohol–sodium alginate, the removal of prometryn (100 mg/kg) in soil was examined. According to the study, prometryn degradation in control samples increased gradually over 30 days before stabilizing at about 20%. On the other hand, samples treated with immobilized beads showed a marked increase in degradation over time, with 70.4% of the initial prometryn eliminated by day 60 [55].

To degrade the fungicide tebuconazole, an investigation was carried out using the bacterial strain *Alcaligenes faecalis* WZ-2, which was immobilized in wheat straw biochar. The results revealed that the immobilized bacterial strain reduced the half-life of the fungicide to 13.3 days, compared with 40.8 days in the control after 60 days of incubation [70]. To degrade residues of fipronil from polluted soil a bacterial strain *Bacillus* sp. FA4 was immobilized in agar and sodium alginate. This study concluded that immobilized FA4 cells with SA and agar disc beads revealed high degradation with reduced half-lives of 7.83 and 7.34 days, respectively [71]. An extensive analysis revealed that, initially, immobilizing degrading bacteria enhanced cells’ resistance to harmful substances and secondly, the immobilized carrier served as a sort of buffer between pollutants and degrading bacteria, preventing direct contact between cells and high pesticide concentrations [72]. However, all studies proved that immobilization technology performs more efficiently for the remediation of various pollutants and SA is a fundamental carrier material that is used in the immobilization process.

### 3.8. Degradation of Chlorothalonil in Tomato Vegetated Soil

**Figure 8:**
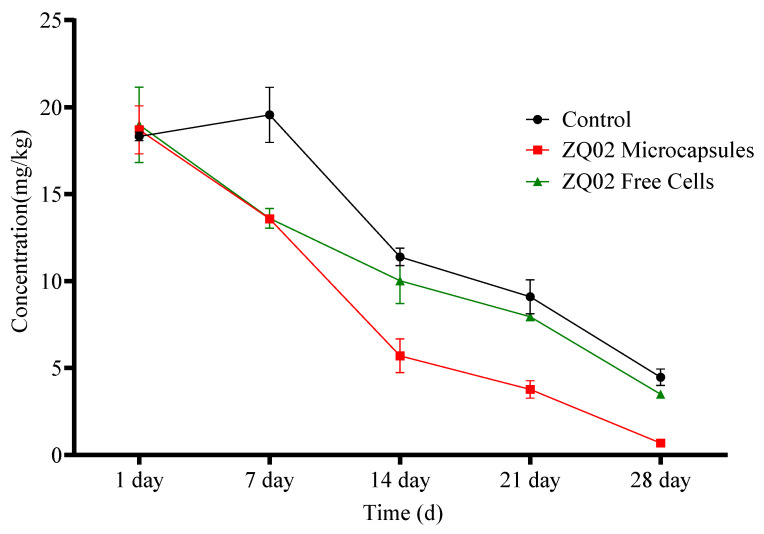
Degradation of chlorothalonil in tomato soil.

Plants have been widely used in research investigating the ecotoxicity of pesticides in soil systems, as well as in allelopathy bioassays. A range of standardized methods, including ISO 11269-1 and ISO 11269-2, ASTM E1963–22, EPA 600/3–88/029, EPS 1/RM/45, ISO 17126, ISO 18763, ISO 29200, ISO 22030, OECD 208, OECD 227, OCSPP 850.4100, OCSPP 850.4230, OCSPP 850.4800, and OPPTS 850.4200, [73,74,75,76,77,78,79,80,81,82,83,84,85,86,87] are currently used to regulate plant-based ecotoxicological assays. These procedures standardize experimental conditions for both greenhouse and laboratory bioassays. Test durations vary widely, ranging from short-term exposure experiments lasting a few hours to long-term assessments extending over several months [88]. Chlorothalonil is extensively applied to control pest infestations in vegetables including tomato and its residues are frequently detected above the maximum residue limits. The accumulation of chlorothalonil residues in tomato causes dietary risks for humans. However, presence of chlorothalonil residue in different parts of tomato plant poses serious threats to non-target organisms. Recently, Zhang et al. [89] reported that the daily intake of chlorothalonil and its degradation products in vegetables are 0.008–0.02 mg/kg, and the acute reference value was settled as 0.03–0.6 mg/kg. However, there is very little information available about the biological remediation of chlorothalonil-contaminated tomato vegetables by microbial species. Figure 8 reveals the biodegradation of chlorothalonil by application of free and encapsulated cells. The initial degradation of chlorothalonil using microcapsules and free cells on day 7 were 28.33% and 27.33%, respectively. Meanwhile, after 28 days, 96.34% and 81.57% of chlorothalonil residues were removed, respectively (Table 3). The distribution of chlorothalonil residues in tomato parts were lower compared to the control and free cells, as shown in Figure 8. Remarkably, a greater amount of chlorothalonil was degraded in tomato parts and soil as a result of the combined action of bacterial microcapsules and the enzymatic reaction of tomato plants. The difference in distribution rate of chlorothalonil in tomato leaves, roots and stem in all parts could be due to variation in plant composition, structure and physiological activities, and survival of applied bacterial cells. Finally, our study explained that, from the field application perspective and dietary risk assessment of non-target organisms, the appropriate interval between chlorothalonil applications should be established for the effective and efficient control of pests and diseases attacking different parts of tomato plants.

In a recent investigation, ZQ02 free cells were used to examine the degradation of chlorothalonil in maize soil. The results showed that the chlorothalonil concentration dropped from 48.73 mg/L to 23.79 mg/L in 7 days in a nutrient culture spiked with 25 mg/L tetracycline. After inoculating soil with ZQ02, the residual chlorothalonil level decreased from 4.92 mg/kg to 0.06 mg/kg in 14 days, lowering the half-life from 9.24 days to 2.35 days [37]. In another study, chlorothalonil hydrolytic dehalogenase enzymes were purified from the bacterial strain *B. subtilis* WB800, and the efficient degradation of chlorothalonil with the concentration of 25 mg/kg in various vegetables was investigated. Results demonstrated that almost all 25 mg/kg of chlorothalonil residues on cherry tomatoes, about 82% of the 45 mg/kg on *Lactuca sativa*, and 67% of the 29 mg/kg on lettuce were eliminated following a 120 min enzymatic treatment. However, chlorothalonil on the other three vegetable types, such as carrot, mushroom, and cauliflower, were degraded by about 40% to 55% [90]. A combination of *B. thuringiensis* strain SG4 and *Bacillus sp*. strain SG2 was used in sodium alginate beads to study the removal of cypermethrin in *Zea mays* plants. The findings showed how *Z. mays* and the bacterial consortium greatly accelerated the breakdown of cypermethrin in soil. In plants treated with 50 mg/kg and 100 mg/kg of cypermethrin, respectively, a 10-fold and 9-fold reduction was observed. A study found usage in this as a biofertilizer to boost crop development on cypermethrin and other pyrethroid-contaminated agricultural land [69]. Another study examined the different bacterial strains’ effects on the breakdown of profenofos in tomato vegetated soil. Results showed that after 9 days, inoculation with strains MUG68 (*Enterobacter kobei*), MUG19 (*Klebsiella oxytoca*), and MUGB6 (*K. aerogenes*) eliminated more than 80% of the profenofos in soil polluted with 150 mg/L. Following a 12-day inoculation with the MUG75 (*E. cloacae*), MUGB24 (*Staphylococcus aureus*), and MUGB12 (*S. aureus*) strains, contaminated soil showed a complete loss of profenofos with a concentration of 150 mg/L [91]. According to our findings, chlorothalonil residues were eliminated from the soil by applying a ZQ02 microcapsule with tomato plants. Microcapsules and tomato plants worked better together than each one alone to reduce chlorothalonil in agricultural soil. Therefore, this method can enhance food quality and soil health without posing environmental risks.

## 4. Conclusions

In this study, the degradation characteristics of *Proteus terrae* strain ZQ02 were evaluated. Strain ZQ02 exhibited a broad degradation spectrum, effectively breaking down chlorothalonil in diverse environments. Encapsulation using sodium alginate as the carrier material resulted in microcapsules that simultaneously degraded chlorothalonil. SEM analysis confirmed that the bacteria were well-fixed and widely distributed within the microspheres. The encapsulated microcapsules demonstrated high mechanical strength, permeability, stability, reusability, and efficient mass transfer. Additionally, the carrier materials played a crucial role in preventing bacterial leakage while shielding the encapsulated bacteria from physical (pH, temperature, humidity) and chemical stressors. Microcapsules significantly enhanced the degradation of chlorothalonil in both soil and tomato plants, outperforming all other treatments, including the control and free-cell ZQ02 treatments. Throughout the study, the microcapsule treatment group exhibited a higher degradation rate than other groups, with significantly greater efficiency than the free-cell bacterial treatment. These findings suggest that immobilization enhances bacterial degradation capacity and accelerates chlorothalonil removal from contaminated environments. The encapsulation technique has substantial theoretical and practical significance for the biodegradation of pesticides, dyes, heavy metals, and other emerging contaminants. Further studies are needed to evaluate the efficiency and adaptability of encapsulated indigenous bacteria under field conditions. The results demonstrated that chlorothalonil-degrading bacterial microcapsules possess stable properties and effective degradation potential, providing a strong technical foundation for the bioremediation of soil and plants, with promising opportunities for further optimization and broader application.

## Figures and Tables

**Table 1:** Orthogonal array configuration design for optimization of microcapsules.

Test No	Con. of CaCl_2_	Con. of SA	Con. of ZQ02	Encap. Time	Regression Equation	*k* ^(h−1)^	*DT*_50_	*R* ^2^
1	3	3	6	15 min	43.07e^−0.64x^	0.64x	1.083	0.999
				12 h	43.67e^−0.381x^	0.381x	1.819	0.982
				36 h	50.44e^−0.365x^	0.365x	1.899	0.993
2	3	2	4	15 min	49.83e^−0.329x^	0.329x	2.106	0.946
				12 h	42.68e^−0.072x^	0.072x	9.627	0.846
				36 h	43.39e^−0.055x^	0.055x	12.602	0.91
3	3	1	2	15 min	41.45e^−0.127x^	0.127x	5.457	0.990
				12 h	41.00e^−0.075x^	0.075x	9.241	0.972
				36 h	43.27e^−0.069x^	0.069x	10.04	0.958
4	2	3	6	15 min	57.60e^−0.6x^	0.6x	1.55	0.91
				12 h	43.41e^−0.095x^	0.095x	7.296	0.949
				36 h	45.04e^−0.061x^	0.061x	11.363	0.969
5	2	2	4	15 min	49.48e^−0.361x^	0.361x	1.920	0.961
				12 h	42.61e^−0.09x^	0.09x	7.70	0.924
				36 h	43.08e^−0.042x^	0.042x	16.503	0.750
6	2	1	2	15 min	46.29e^−0.245x^	0.245x	2.829	0.972
				12 h	42.67e^−0.083x^	0.083x	8.351	0.929
				36 h	43.06e^−0.04x^	0.04x	17.32	0.882
7	1	3	6	15 min	52.15e^−0.507x^	0.507x	1.367	0.973
				12 h	61.34e^−0.273x^	0.273x	2.539	0.971
				36 h	43.35e^−0.056x^	0.056x	12.377	0.926
8	1	2	4	15 min	47.57e^−0.426x^	0.426x	1.627	0.982
				12 h	41.88e^−0.11x^	0.11x	6.301	0.993
				36 h	42.84e^−0.058x^	0.058x	11.950	0.944
9	1	1	2	15 min	47.54e^−0.397x^	0.397x	1.745	0.982
				12 h	43.41e^−0.113x^	0.113x	6.134	0.986
				36 h	43.07e^−0.057x^	0.057x	12.160	0.950

**Table 2:** Kinetic models of chlorothalonil biodegradation in soil.

Concentration of Chlorothalonil	Sample Name	Kinetic Model	Rate Constant (*k*)	Half-Life (t ½)	*R*^2^ Batch	Degradation (%)
30 mg/kg	Control	y = 27.375e^−0.013x^	0.013	53.319	0.98	25.03
	Treatment	y = 31.545e^−0.127x^	0.127	5.457	0.92	94.73
20 mg/kg	Control	y = 18.031e^−0.021x^	0.021	33	0.97	26.07
	Treatment	y = 20.512e^−0.227x^	0.227	3.05	0.97	96.02
10 mg/kg	Control	y = 8.975e^−0.029x^	0.029	23.901	0.99	18.89
	Treatment	y = 9.119e^−0.373x^	0.373	1.858	0.98	92.17

**Table 3:** Kinetic models of chlorothalonil biodegradation in tomato-planted soil.

Concentration of Chlorothalonil	Sample Name	Kinetic Model	Rate Constant (*k*)	Half-Life (t ½)	*R*^2^ Batch	Degradation (%)
	Control	y = 23.538e^0.053x^	0.053	13.07	0.84	75.61
20 mg/kg	ZQ02 Free Cells	y = 21.265e^−0.058x^	0.058	11.95	0.97	96.34
	ZQ02 Microcapsules	y = 27.171e^−0.117x^	0.117	5.92	0.94	81.57

## Data Availability

The original contributions presented in this study are included in the article. Further inquiries can be directed to the corresponding authors.

## Supplementary Materials

The following supporting information can be downloaded at: https://www.mdpi.com/article/10.3390/toxics14050352/s1, Figure S1: Growth curve of the bacterial strain ZQ02 showing optical density (OD_600_) over time (0–48 h) under controlled incubation conditions; Figure S2: Preparation of microcapsules. Left hand sided is formation of microcapsules with 3% SA, 2% CaCl_2_ and 60 g/L of wet mas of ZQ02 and right hand sided is microcapsules lacking ZQ02 wet calls and just contained 3% SA and 2% CaCl_2_.
