# Supplementary material for: Microbial Biodegradation of Chlorothalonil Residual Pollutants in Soil and Tomato Plants by Microencapsulated Proteus terrae ZQ02
Source: Toxics. 2026 Apr 22;14(5):352. doi: 10.3390/toxics14050352 (PMC13210798; doi:10.3390/toxics14050352)
Supplement: Supplementary file 1 [file toxics-14-00352-s001.zip › toxics-4240673-supplementary.pdf]

Microbial Biodegradation of Chlorothalonil Residual Pollutants in Soil and Tomato Plants by Microencapsulated *Proteus terrae* ZQ02

Sajjad Ahmad [1], Jie Liu [1,*] and Murugesan Chandrasekaran [2,*]

[1] College of Plant Protection, South China Agriculture University, Guangzhou 510642, China; iamdrsajjad@gmail.com ORCID 0000-0002-6001-5664

[2] Department of Food Science and Biotechnology, Sejong University, 209 Neundong ro, Gwangjin gu, Seoul 05006, Republic of Korea ORCID 0000-0002-8520-252X

* Correspondence: jieliu@scau.edu.cn (J.L.); chandrubdubio@sejong.ac.kr (M.C.)

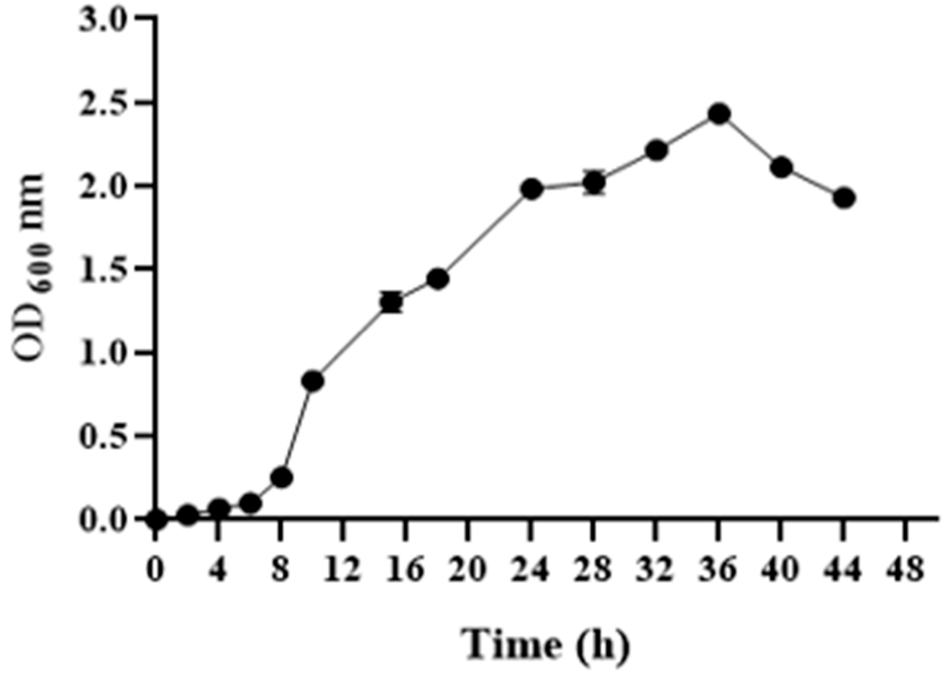

Figure S1 Growth curve of the bacterial strain ZQ02 showing optical density (OD_{600}) over time (0–48 h) under controlled incubation conditions.

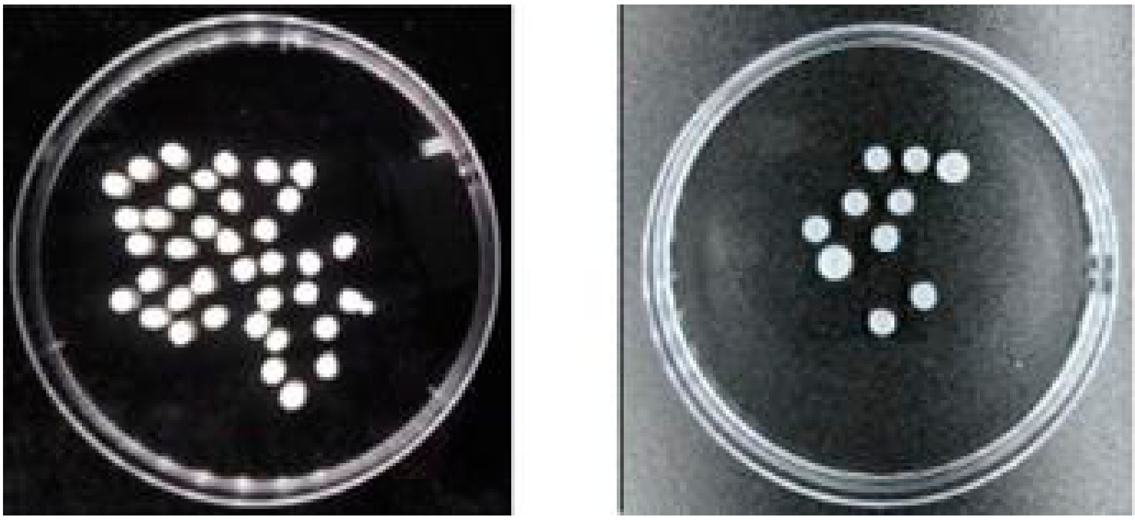

Figure S2. Preparation of microcapsules. Left hand sided is formation of microcapsules with 3% SA, 2% $CaCl_2$ and 60 g/L of wet mas of ZQ02 and right hand sided is microcapsules lacking ZQ02 wet calls and just contained 3% SA and 2% $CaCl_2$.
